# Guided Frontal Sinus Osteotomy: A Pilot Study of a Digital Protocol for “In-House” Manufacturing Surgical Cutting Guides

**DOI:** 10.3390/jcm14093141

**Published:** 2025-05-01

**Authors:** Antonio Romano, Stefania Troise, Raffaele Spinelli, Vincenzo Abbate, Giovanni Dell’Aversana Orabona

**Affiliations:** Maxillofacial Surgery Unit, Department of Neurosciences, Reproductive and Odontostomatological Science, University of Naples Federico II, 80138 Naples, Italy; romano.antonio1972@gmail.com (A.R.); raffaele.spinelli.1991@gmail.com (R.S.); vincenzo.abbate@unina.it (V.A.); giovanni.dellaversanaorabona@unina.it (G.D.O.)

**Keywords:** 3D printing, CAD/CAM, frontal sinus surgery, patient-specific modeling, stereolithography, surgical cutting guides

## Abstract

**Objective:** Frontal sinus surgery is still challenging for surgeons; the frontal osteotomy with the preparation of a frontal bone flap to access the sinus is usually hand-crafted by experienced surgeons. The objective of our study is to present a fully digital protocol for the manufacturing of “in-house” surgical cutting guides, customized to the patient’s anatomy, to perform precise frontal sinus osteotomy, showing the costs, times, and intraoperative complications reduction. **Materials and Methods:** A prospective study was conducted on 12 patients with complex pathologies involving the frontal sinus who underwent frontal sinus osteotomy in the Maxillofacial Surgery Unit of the Federico II University of Naples, from January 2021 to April 2025, considering the last surgery in November 2023. The same digital protocol to manufacture the surgical cutting guide was used for all the 12 patients. The first step was to upload the preoperative CT images in DICOM format to the software Mimics Medical to perform a rapid segmentation of the skull region of interest to create a 3D object and to identify the frontal sinus margins and the osteotomy lines. The second step was to realize the surgical cutting guide, incorporating the design of titanium plates to fix onto the skull in order to make a precise osteotomy. The final digital step was to export the cutting guide 3D object in the software “Formlab-Form 3B” to print the model with a specific resin. The model was then used during the surgery to perform the precise frontal osteotomy by piezo surgery. The clinical outcomes, in terms of complications and recurrences, were then recorded. **Results:** In all the patients, no intraoperative complications occurred; the median follow-up was 31.7 months and at one year of follow-up only one patient experienced a recurrence. The mean operative time was about 4 h, with a frontal osteotomy time of about 23 min. Digital protocol time was about 4 h while printing times were between 2 and 4 h. **Conclusions:** This “in-house” protocol seems to demonstrate that the use of intraoperative templates for the realization of the frontal sinus osteotomy reduces preoperative and intraoperative costs and times, reducing the risk of intraoperative complications, and also allows less experienced surgeons to perform the procedure safely. Obviously, this study is to be considered a “pilot study”, and other studies with large cohorts of patients will have to confirm these promising results.

## 1. Introduction

The management of frontal sinus pathologies currently remains a challenge for surgeons, and the correct choice of the most suitable surgical approach is still a discussed topic in the literature. Despite the evolution of advanced endoscopic techniques for frontal sinus surgery, the open approach continues to be a valid alternative in cases of difficult management only with the endoscopic approach [1,2,3]. Chiu et al. [4] in 2005 had identified the conditions in which, for example, benign tumors as osteomas of the frontal sinus required a double endoscopic and open approach for the correct surgical management. In general, the currently accepted indications for a frontal open osteoplastic include chronic frontal sinusitis after failed endonasal surgery, muco(pyo)cele, involvement of the posterior wall or anterior wall erosion with fistulization, severe frontal bone fractures, and severe odontogenic infections which result in orbital cellulitis and frontal involvement and tumors [5,6,7,8,9], as well as cosmetic surgery procedures such as facial feminization [10,11].

Over the years, the frontal bone flap has been harvested by freehand due to the surgeon’s experience, resulting in a procedure that could only be performed by expert surgeons in the field. The advent of virtual surgical planning and CAD/CAM (computer-aided design/computer-aided manufacturing) technologies, ensuring the design of the surgical procedure down to the smallest details, has become a valid support even for younger and less experienced surgeons. In particular, the possibility of manufacturing patient-specific surgical cutting templates, designed on the patient’s anatomy, and therefore performing a “guided surgery” is a valid tool also in modern cranio-maxillo-facial surgery [12,13,14]. In fact, the use of cutting templates is now employed daily with satisfactory results in oncology [15,16], traumatology [17,18], orthognathic surgery [19,20], and oral surgery and implantology [21,22,23,24]. Cutting guide manufacturing is usually carried out by engineers or by surgeons in collaboration with engineers, basing the project on patient’s preoperative computer tomography (CT) images. After the virtual planning, the cutting guide is prototyped and realized in specialized laboratories/industries. This process, usually, can lengthen patient waiting time; in fact, based on the complexity of the case, the industry requires a lead time of at least seven to ten working days, during which one to three online meetings are held to discuss the planning. Furthermore, depending on the complexity of the cases, the costs recorded for each custom-made template range between 2000.00 and 8000.00 USD [25,26]. Hence, the need to realize “in-house” cutting templates that, on the one hand, guarantee the precision and accuracy of guided surgery but, on the other hand, do so at low costs and in a timely manner for the hospital and the patient. Thus, the aim of our study is to present a digital protocol for manufacturing fully “in house” surgical cutting guides for frontal sinus osteotomy, from design to 3D printing up to employment in the operating room, highlighting how intraoperative use of these guides can reduce operative time and avoid possible intraoperative complications. Given the small sample size of 12 patients, this study should be considered a pilot study, and future studies with a larger cohort of patients should confirm the promising results of this pilot study.

## 2. Materials and Methods

This work is a prospective study on 12 patients who underwent frontal sinus osteotomy in the Maxillofacial Surgery Unit of the Federico II University of Naples, from January 2021 to April 2025, considering the last surgery in November 2023 to guarantee a minimum follow-up of 18 months. Ethical review and approval for this study was obtained by the Ethics Committee of the University Federico II of Naples with the protocol number 32/2024.

Inclusion Criteria

The patients enrolled in the study satisfied the following inclusion criteria:

- Complex benign pathologies involving the frontal sinus (osteomas, mucoceles, recurrent sinusitis), needing a frontal sinus osteotomy + ESS (DRAF procedures) according to Chiu classification [4];
- Maxillofacial computer tomography (CT) performed within 7 days before surgery;
- Intraoperative employment of the “in-house” manufactured surgical cutting guide;
- A minimum follow-up of 18 months.

Exclusion Criteria

The patients who did not satisfy the inclusion criteria or presented the following exclusion criteria were not enrolled in the study:

- Contraindications to perform a frontal sinus osteotomy (pathologies resolved with the only endoscopic approach);
- Malignant tumors;
- Previous traumas/chemotherapy/radiotherapy in the frontal region;
- Patient refusal of surgery;
- Insufficient follow-up.

### 2.1. Data Collection

All the patients underwent a presurgical clinical examination and a preoperative CT. The following data were collected: sex, age, type of diseases, symptomatology, and outcomes. All the patients were informed about the surgical procedure and the employment of a cutting guide and signed the informed consent.

### 2.2. Step-By-Step Surgical Cutting Guides Manufacturing Protocol

The CAD/CAM-based workflow protocol was performed by the same three medical doctors (AR, ST, and RS) without the intervention of engineers, using the technological equipment available in the hospital, and was the following:

#### 2.2.1. Conversion from DICOM (Digital Imaging and Communications in Medicine) Files to STL (Stereolithography) Files

The first step was to upload the preoperative CT images with a slice thickness of 0.5 mm in DICOM format to the software Mimics Medical (Mimics^®^ 17.0, Materialise; Leuven, Belgium) to perform a rapid segmentation of the skull region of interest, ensuring high-resolution data for optimal model accuracy. A standard bone mask was created using the pre-set threshold values (226–3071 Hounsfield Units), eventually separating the osteoma/mucocele from the surrounding bone tissue or to better delineate very thin anatomical structures. Once the segmentation was completed, the mask was converted into a 3D object. A cutting plane was then applied to remove unnecessary portions of the scan, such as the lower two-thirds of the face and the posterior part of the cranium, in order to reduce the file size and simplify subsequent processing. At the end of the procedure, the 3D model was converted into a stereolithography (STL) file and exported in an open-source software, Meshmixer 3.5 (Autodesk, Inc©, San Rafael, CA, USA).

#### 2.2.2. Identification of the Frontal Sinus Margins and Selection of Osteotomy Lines

**Figure 1:**
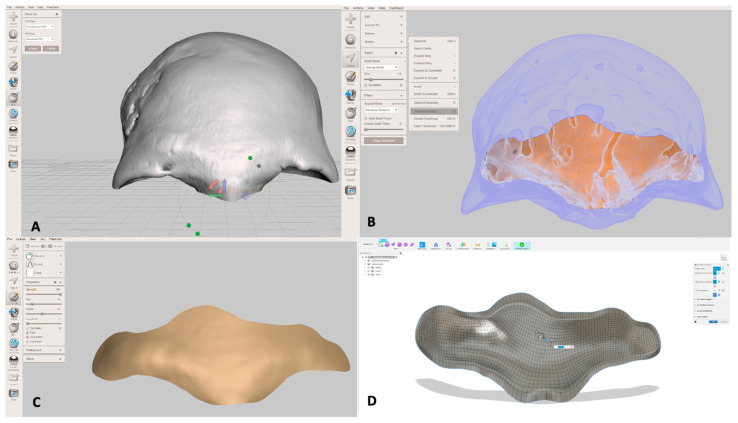
Computer-Aided Design (CAD) of surgical cutting guide. (**A**) Segmentation of the skull; (**B**) identification of the frontal sinus osteotomy lines; (**C**) realization of the 3D object of the frontal sinus anterior wall; (**D**) conversion of the 3D object in the 3D surgical cutting guide.

Through the “plane cut” and “make solid” functions of the Meshmixer 3.5 software, the 3D image was enhanced in order to convert the surface into a solid volume. At this point, the area corresponding to the frontal sinuses was identified in transparency, and with the “brush” function and the “smooth Boundary” function the osteotomy area was highlighted taking care to avoid the bony septa and reliving the cut in empty cavity to facilitate the procedure. The final step was to extract the resulting surface, smoothing the edges with the function “Robust Smooth” and exporting the final object into Fusion 360 software (Autodesk, Saulito, CA, USA, 2023) (Figure 1).

#### 2.2.3. Cutting Guide Design

**Figure 2:**
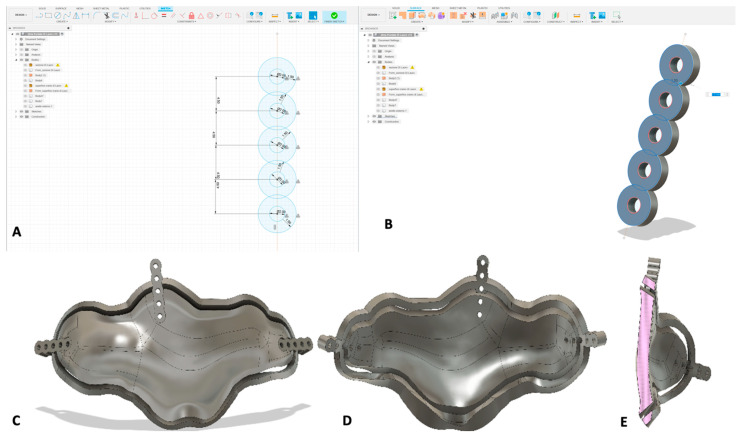
Implementation of the titanium plate model into the cutting guide design. (**A**) Calculation of plate holes diameter; (**B**) realization of the 3D object of a plate with 5 holes; (**C**) final 3D object of the guide, implemented with plates, in the frontal view, (**D**) posterior view, and (**E**) lateral view.

Using Fusion 360’s “Quad Remesher” extension, the resulting object was converted into T-Spline. This conversion was necessary because T-Spline geometry allows for easier and more flexible editing compared to mesh models, which limit the available tools and actions within the CAD environment. Using the “thicken” function, the surface was transformed from a surface into a solid object, with a thickness varying between 2 and 3 mm depending on the local anatomical shape. Smaller guides were assigned a greater thickness to ensure sufficient mechanical strength, whereas larger guides required less thickness to maintain a balance between robustness and flexibility, optimizing handling and fitting during surgery. The “section analysis” function was used to generate cross-sectional views of the surgical guide, enabling a detailed evaluation of both internal and external geometries during the design process. Although this function does not directly modify the geometry, it is essential for accurately identifying the areas where a bevel must be manually created on the guide’s surface. By using the section views as a reference, an inclined osteotomy surface was modeled, allowing the bone cut to be performed along a sloped plane rather than a perpendicular one. Typically, an inclination of approximately 30° was applied to the cutting surface. However, in the presence of prominent frontal septa, the bevel angle was increased up to 50° to sufficiently weaken these bony structures and facilitate their controlled osteotomy during surgery. This inclination facilitates the repositioning of the bone segment and prevents it from sinking into the frontal sinus. The inclination values were defined based on accumulated surgical experience and anatomical considerations, with the postoperative outcome evaluation guiding further optimization of cutting guide designs. The surgical template already incorporated the design of titanium plates that will be used to fix the guide to the skull, ensuring stable positioning and enabling a highly precise osteotomy. The integration of fixation elements within the template design reduces intraoperative time and avoids potential inaccuracies from separate plate positioning. Using the “Create Sketch” function, the shape of the plate was reproduced, carefully setting the hole size and the distance between hole centers to not only match the screw diameter but also to guarantee full compatibility with the fixation system (plates and screws) intended for clinical use (Figure 2). At the end of this step, the final object was converted into STL files and exported to the software “Formlab-Form 3B” (Formlabs Form 3B+, located in Somerville, MA, USA) for 3D printing using biocompatible surgical guide resin (Formlabs), classified as a Class I medical device and specifically designed for surgical applications.

#### 2.2.4. Rapid Prototyping 3D Printing

**Figure 3:**
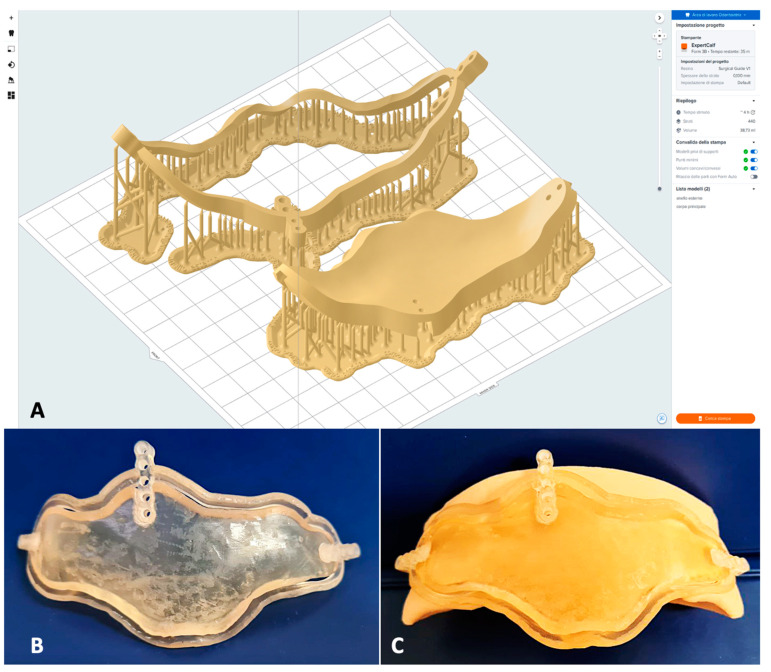
Realization of the guide resin model from computer-aided manufacturing (CAM) to 3D printing. (**A**) Set up of the surgical guide 3D object with the supports in the Formlabs printing software; (**B**) final resin model of the surgical guide; (**C**) fitting of the guide on a patient’s skull model.

Using the software “Formlab-Form 3B” 3D printer, supports were set to obtain the highest possible quality of the model. (Figure 3A) A resin stereolithography apparatus (SLA) 3D printer was employed for rapid prototyping; the selected material was the Formlab resin surgical guide V1 (Formlabs, Somerville, MA, USA). Once the print was completed, the model was washed in isopropyl alcohol using the FormWash (Formlabs Somerville, MA, USA) device for 20 min and photopolymerized using the FormCure (Formlabs Somerville, MA, USA) device for other 20 min. The final model is shown in Figure 3C.

### 2.3. Surgical Procedure (Employment of Template)

**Figure 4:**
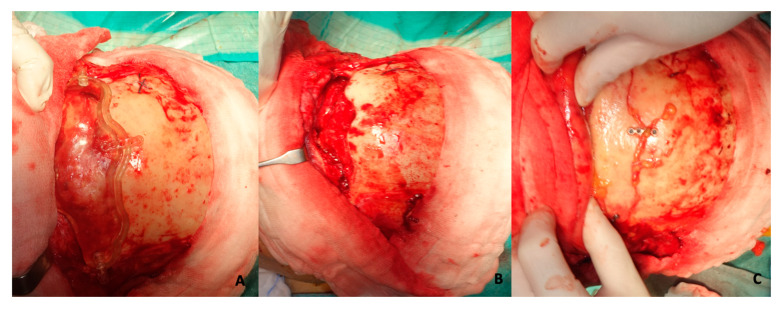
Surgical employment of cutting guide in order to perform a precise frontal osteotomy. (**A**) Fitting and fixation of the guide on patient’s skull; (**B**) final realized precise osteotomy; (**C**) repositioning of frontal bone bandeaux on skull with titanium plates and screws.

The surgery was performed under general anesthesia with an orotracheal intubation by the same anesthesiologist and surgical team. All the patients underwent a double procedure, an endoscopic sinus surgery (ESS) and an open coronal approach, according to the extension of the disease. In all patients, an anterior and posterior ethmoidectomy was performed to clearly visualize the portion of the pathology in continuity with the medial wall of the orbit and a Draf II for a thorough cleaning and removal of the frontal mucosa. After the endoscopic treatment of the involved sinuses, the coronal flap was performed, harvesting a galea flap to use after for the frontal obliteration; after the exposure of the skull bone, the surgical cutting guide was placed on the anterior frontal board until a perfect fit was achieved, and then fixation was performed using titanium screws. Osteotomy was performed by piezosurgery with an inclination of 45° following the margin of the template until the frontal bandeau was detached through the use of thin chisels. In this way, the planning was followed to avoid damage to structures external to the frontal sinus. After the frontal sinus cleaning procedures, an abdominal fat graft was harvested and was used with the rotated galea flap to perform the frontal obliteration and to isolate the frontal sinus. The frontal bone bandeau was cleaned from residual frontal mucosa and was repositioned and fixed with titanium plates and screws, using the holes previously used. A surgical procedure example is shown in Figure 4. The surgical application of the cutting guide is explicated in the Video S1.

After the surgical procedure, all the patients underwent antibiotic therapy for 7 days and daily dressings.

### 2.4. Follow-Up and Outcomes

All the patients underwent a postsurgical clinical examination and a postoperative CT. The minimum follow-up was 17 months after surgery. The complications related to the procedures were evaluated intraoperatively at the one-month and one-year outpatient controls and infections, hypertrophic scars, rhinocerebrospinal fistulas, fractures, recurrences, and mucoceles were recorded.

## 3. Results

### 3.1. Population Features

Twelve patients were treated for complex diseases involving the frontal sinus, performing a frontal sinus osteotomy using a fully “in-house” surgical cutting guide. The main features of the 12 patients are shown in Table 1. The sex ratio was 4:1 M–F and the average age was 56.5 years (range 31–72 years). The causes of surgery were frontal-ethmoidal mucocele in the 41.7% of cases (5/12), recurrent frontal sinusitis in 33.3% of cases (4/12), and frontal-orbital-ethmoidal osteoma in 25% of cases (3/12). The symptoms reported by the patients were as follows: headache and pain (50%), rhinorrhea (41.7%), frontal wall erosion with skin fistulization (16.7%), lateral globe dislocation (16.7%), hyposmia (8.3%), exophthalmos, and dystopia (8.3%), and no symptoms (8.3%). In all 12 cases, the mean operative time was about 4 h, considering an average time of procedure of about 30 min. The mean time of the entire procedure was about 4.5 h, while the mean time for the frontal osteotomy procedure was about 23 min, including the time to re-fix the frontal bone with plates and screws at the end of the procedure. No patient reported intraoperative complications related to the guided frontal osteotomy.

**Figure 5:**
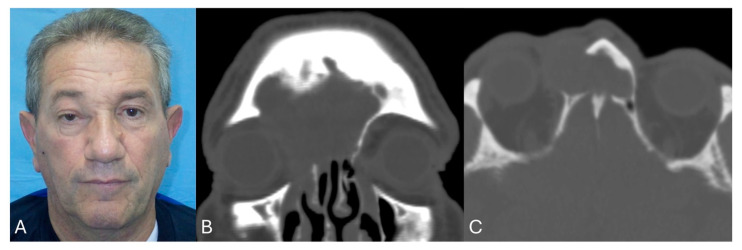
Case example pre-surgery. (**A**) Clinical photo with evidence of dystopia and globe dislocation; (**B**) pre-operative CT coronal view and (**C**) pre-operative CT axial view, with evidence of frontal and orbital involvement by mucocele.

**Figure 6:**
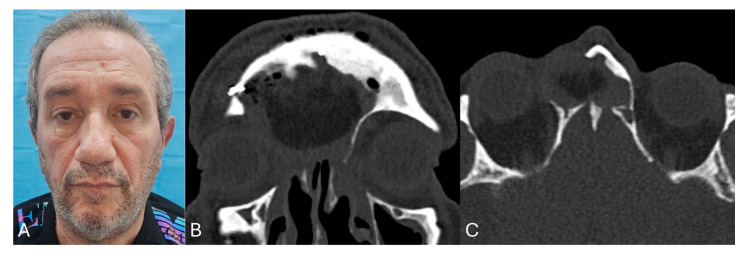
Case example post-surgery. (**A**) Clinical photo with evidence of resolution of dystopia and globe dislocation; (**B**) post-operative CT coronal view and (**C**) post-operative CT axial view.

The average follow-up after surgery was about 31.7 months (range 18–50 months). Regarding the outcomes, considering the 11 symptomatic patients, 63.6% had a total resolution of the symptoms, while 27.3% a partial resolution. Only 1 patient of the 12 (8.3%) reported recurrence after 1 year, with the related symptoms. A case example with clinical and radiological images is presented in Figure 5 and Figure 6.

### 3.2. Cutting Guides Manufacturing Results

The digital data acquisition and the surgical guide design times were approximately 4 h. Printing times were between 2 and 4 h according to the guide’s dimensions. An average of 25 mL of resin was used and the total costs for consumables were about EUR 5 per piece, considering the initial cost for the 3D printer Formlabs-form 3B+ and all related software and tools is approximately 15,000.00 euros.

### 3.3. Video

Video1 describes the surgical steps of positioning and fixating the cutting guide, the simplified frontal osteotomy and, at the end of frontal sinus washing, the repositioning of the frontal bone bandeaux with titanium plates and screws.

## 4. Discussion

The introduction of a “guided surgery”, based on the use of highly patient-specific CAD/CAM technology, has completely revolutionized the cranio-maxillo-facial surgery, especially with regard to hard tissues [12,13,14]. Virtual surgical planning of the intervention and the application of the project directly in the operating room allows the surgeon to increase precision and effectiveness and reduce operating times in various fields of cranio-maxillo-facial surgery [15,16,17,18,19,20,21,22,23,24]. However, this procedure has some limitations: the production times of these cutting templates, which must be produced by specialized industries, ranges from 7 to 10 days; the direct and indirect costs, which for each template amount to between USD 2000 and 8000, depending on the size and complexity; the need for engineers who work in the hospital or alternatively who follow the case remotely with webinars and programming [25,26]. To overcome these limitations, many hospitals have developed “in-house” protocols for the creation of surgical templates and guides to shorten manufacturing times and reduce costs, although there is an initial investment cost for 3D equipment and software. The introduction of an in-house digital workflow for manufacturing 3D printed surgical cutting guides represents a advancement in the management also of frontal sinus pathologies. As McAllister wrote in his article [27], this approach addresses several key challenges by reducing patient wait times and enhancing surgical precision. In fact, by manufacturing the surgical guide in house, waiting times from the laboratory to the hospital are avoided, which is essential especially in symptomatic patients [28,29,30].

Considering these premises, the aim of our study is to present a digital protocol for manufacturing fully “in house” surgical cutting guides for frontal sinus osteotomy, from design to 3D printing up to employment in the operating room, highlighting how intraoperative use of these guides can reduce operative time and avoid possible intraoperative complications, as well as that they can reduce production costs.

First of all, the total production time for each template, from design to 3D printing, amounts to about 4–6 h, depending on the complexity of the case. The planning is performed by surgeons passionate about CAD/CAM technologies, without the intervention of engineers. This allows the CT scan to be performed as soon as possible, without risking that the pathology could compromise the patient’s anatomy in the time between the CT scan and the manufacturing of the template by an external industry.

The literature reports cases of frontal flap harvesting, lasting up to 2 h without specifying the time for the only osteotomy [31,32]; in our cases, employing surgical cutting guides, the time for osteotomy and fixation at the end of the procedure was about 23 min (range 20–28 min). Moreover, the planning of a surgical guide allows the surgeon to avoid the most difficult areas to osteotomize, such as the thickest areas or intra-sinus septa; in this way, less experienced surgeons can also perform this procedure. Furthermore, this system allows us to identify the areas in which the bone cortex is thicker and, therefore, more difficult to penetrate through piezosurgery. In this way, the frontal osteotomy is easier and safer, avoiding important complications reported in the literature such as breakage of the frontal bandeau and piezo cutters, or injuries to the surrounding tissues [33,34,35].

Historically, in the pre-CAD/CAM era, Weber et al. [36] reported in their study that the most frequent complication in frontal bony flap harvesting was injury of the periorbita with the exposure of orbital fat and consequent orbital hematoma with “blue eye”; other recorded complications were fracture of the anterior wall (21.3%), frontal bony flaps too small to permit complete mucosal removal (8.5%) requiring removal of additional parts of the frontal bone, and frontal bony flaps too large (8.5%) with consequent injury to the dura or the superior sagittal sinus. Certainly, with the modernization of surgical techniques and the advent of virtual surgical planning, many of these complications are no longer reported, but unfortunately even in 2025 there is the risk of incurring intraoperative problems, as reported in the very recent article by Rampinelli et al. [37].

In our sample, by performing a virtual surgical planning with a guided frontal osteotomy no intraoperative complications occurred.

Lee et al. [5] reported in their review of the literature a symptomatic improvement of 85% with a complications rate of 19.4% without guided osteotomy. In our sample with guided frontal osteotomy, the symptomatic improvement rate was 91.7%, with only one case (8.3%) of recurrence of pathology and related symptoms. In 27.3%, the partial resolution of the symptoms refers to chronically established conditions such as hypo/anosmia or conditions such as dislocation of the globe, in which chronic alteration of the patient’s basic anatomy delays complete recovery of the physiological position of the eye. The case example shows the resolution of the orbital symptoms with good healing of the tissues and the partial recovery of the physiological position of the globe.

Another interesting aspect to consider is the reduction in direct and indirect production costs. As mentioned, the cost for a surgical guide produced by an external industry is between USD 2000.00 and 8000.00, depending on the case [25,26]. If this cost is considered for 12 cases, our sample, an expense of USD 24,000.00–96,000.00 might have been necessary. The “in-house” protocol is a very low-cost method. In fact, the initial expense of 15,000.00 euros for the printer and the dedicated software is convenient to save on a single piece. The cost for a single case was estimated to be around 5 euros. In fact, approximately 25 mL of resin was required to make each 3D model, costing approximately EUR 5.00 per unit, safeguarding hospital expenses and positively impacting on the department’s economic indicators [38].

Furthermore, while previously this surgery was only the prerogative of expert surgeons, with the support of these technologies even younger surgeons can approach these procedures [39]. In accordance with Abo Sharkh [40], the use of these cutting guides assists even less experienced surgeons in avoiding bony septa and thicker cortical areas and accessing more difficult spaces, thereby improving overall surgical outcomes and reducing the risk of intraoperative complications such as iatrogenic fractures, breakage of the frontal bone flap, breakage of the piezo burs, and rhinoliquor fistulas.

Despite the encouraging results and premise, several limitations of this study must be underlined. First of all, the small sample of patients with a gender disparity; for this reason, the authors want to underline the pilot intention of this study that could play the role of a founder study for subsequent multicenter research with larger samples. Other limitations are the slow learning curve of software use and the availability of tools. Moreover, the lack of a control group does not allow us to compare the precise time for performing only the osteotomy and repositioning and fixation of the frontal flap because this time is not usually reported in the literature. However, based on the experience of surgeons, the authors of this paper, the use of the surgical template provides greater comfort that translates into a reduction in operating times compared to their experience, although it is not quantifiable. Anyway, the preliminary results of this protocol are promising, considering strengths and advantages such as a as reduction in operating times, reduction in the risk of intraoperative complications, comfort for even less experienced surgeons, and limited costs without the involvement of external laboratories and companies.

Moreover, another strength of this work is the possibility to reproduce the protocol in other fields, for example, in cosmetic surgery, in particular in facial feminization procedures and the reduction in frontal bumps, and for highly personalized surgery.

## 5. Conclusions

The introduction of a comprehensive “in-house” digital workflow for the design and manufacturing of personalized cutting guides represents an advancement in the performing of frontal sinus osteotomy for benign pathologies. This approach allows us to reduce surgical times, increase the accuracy of osteotomies, and decrease the risk of intraoperative complications, allow less experienced surgeons to perform the procedure safely, speed up the prototyping process, and reduce production costs. Obviously, this study is to be considered a “pilot study”, and other studies with large cohorts of patients will have to confirm these promising results.

## Figures and Tables

**Table 1:** Main features of the 12 enrolled patients.

Case	Sex	Age	Type of Disease	Signs and Symptoms	Time of Procedure *	Follow-Up	Complications	Outcomes
1	M	47	Frontal-ethmoidal mucocele	Frontal wall erosion with skin fistulization	28 min (about 5 h)	50 months	No	Resolution of the symptom
2	M	68	Recurrent frontal sinusitis	Headache, Rhinorrhea	26 min (about 5.5 h)	46 months	No	Resolution of the symptom
3	M	53	Frontal-ethmoidal mucocele	Headache, pain	25 min (about 4 h)	43 months	No	Resolution of the symptom
4	M	31	Frontal-orbital-ethmoidal osteoma	Lateral globe dislocation	23 min (about 5.5 h)	38 months	No	Partial resolution of the symptom
5	F	72	Recurrent frontal sinusitis	Pain, rhinorrhea, and hyposmia	22 min (about 5 h)	36 months	No	Partial resolution of the symptom (hyposmia)
6	M	43	Frontal-ethmoidal mucocele	Headache, pain	25 min (about 4.5 h)	30 months	No	Resolution of the symptom
7	F	57	Frontal-orbital-ethmoidal osteoma	Exophthalmos, dystopia, and lateral globe dislocation	20 min (about 5 h)	28 months	No	Partial resolution of the symptom
8	M	67	Frontal-ethmoidal mucocele	Headache, pain, rhinorrhea, and frontal wall erosion with skin fistulization	25 min (about 4.5 h)	26 months	Recurrence after one year	Headache, pain, and rhinorrea
9	M	71	Recurrent frontal sinusitis	Headache, pain, and rhinorrhea	23 min (about 4 h)	24 months	No	Resolution of the symptom
10	M	48	Frontal-ethmoidal osteoma	Asymptomatic	20 min (about 5 h)	21 months	No	/
11	F	58	Recurrent frontal sinusitis	Headache, pain, and rhinorrhea,	21 min (about 4 h)	20 months	No	Resolution of the symptom
12	M	63	Frontal-ethmoidal mucocele	Frontal wall erosion with skin fistulization	22 min (about 4 h)	18 months	No	Resolution of the symptom

* Complete frontal sinus osteotomy procedure.

## Data Availability

Available upon request to the corresponding author.

## Supplementary Materials

The following supporting information can be downloaded at: https://www.mdpi.com/article/10.3390/jcm14093141/s1, Video S1: Description of the surgical steps of positioning and fixating the cutting guide, the simplified frontal osteotomy and, at the end of frontal sinus washing, the repositioning of the frontal bone bandeaux with titanium plates and screws.

## Abbreviations

The following abbreviations are used in this manuscript:

CAD/CAM	Computer-Aided Design/Computer-Aided Manufacturing
CT	Computer Tomography
3D	Dimensional
VSP	Virtual Surgical Planning
USD	Dollars
ESS	Endoscopic sinus surgery
DICOM	Digital imaging and communication in medicine
STL	Standard triangulation language
SLA	Stereolithography Apparatus
EUR	Euros
